# Robustness of neuronal tuning to binaural sound localization cues against age-related loss of inhibitory synaptic inputs

**DOI:** 10.1371/journal.pcbi.1009130

**Published:** 2021-07-09

**Authors:** Go Ashida, Daniel J. Tollin, Jutta Kretzberg

**Affiliations:** 1 Cluster of Excellence "Hearing4all", Department of Neuroscience, University of Oldenburg, Oldenburg, Germany; 2 Department of Physiology and Biophysics, University of Colorado School of Medicine, Aurora, Colorado, United States of America; University of Pittsburgh, UNITED STATES

## Abstract

Sound localization relies on minute differences in the timing and intensity of sound arriving at both ears. Neurons of the lateral superior olive (LSO) in the brainstem process these interaural disparities by precisely detecting excitatory and inhibitory synaptic inputs. Aging generally induces selective loss of inhibitory synaptic transmission along the entire auditory pathways, including the reduction of inhibitory afferents to LSO. Electrophysiological recordings in animals, however, reported only minor functional changes in aged LSO. The perplexing discrepancy between anatomical and physiological observations suggests a role for activity-dependent plasticity that would help neurons retain their binaural tuning function despite loss of inhibitory inputs. To explore this hypothesis, we use a computational model of LSO to investigate mechanisms underlying the observed functional robustness against age-related loss of inhibitory inputs. The LSO model is an integrate-and-fire type enhanced with a small amount of low-voltage activated potassium conductance and driven with (in)homogeneous Poissonian inputs. Without synaptic input loss, model spike rates varied smoothly with interaural time and level differences, replicating empirical tuning properties of LSO. By reducing the number of inhibitory afferents to mimic age-related loss of inhibition, overall spike rates increased, which negatively impacted binaural tuning performance, measured as modulation depth and neuronal discriminability. To simulate a recovery process compensating for the loss of inhibitory fibers, the strength of remaining inhibitory inputs was increased. By this modification, effects of inhibition loss on binaural tuning were considerably weakened, leading to an improvement of functional performance. These neuron-level observations were further confirmed by population modeling, in which binaural tuning properties of multiple LSO neurons were varied according to empirical measurements. These results demonstrate the plausibility that homeostatic plasticity could effectively counteract known age-dependent loss of inhibitory fibers in LSO and suggest that behavioral degradation of sound localization might originate from changes occurring more centrally.

## Author summary

Accurately locating a sound source is one of the most fundamental functions of the auditory system. Behavioral studies report that sound localization ability generally degrades with aging in both laboratory animals and humans. Anatomical studies in aged animals have observed considerable reductions of inhibitory inputs into the neuronal circuit responsible for sound localization. Paradoxically, physiological studies at the brainstem level, however, find only minor age-related changes in the function of the sound localization circuit. In order to bridge the puzzling discrepancy between these observations, we developed a model of the corresponding neuronal circuit and simulate its function by varying the number of inhibitory afferents. To simply mimic the activity-dependent adjustment of synaptic inputs, we also varied the strength of remaining inhibition. Consistent with the empirical findings, our simulations demonstrate that functional changes of the sound localization circuit remain relatively small, compared to the reduced number of inhibitory inputs. This functional robustness is further enhanced by adjusting the synaptic strength. These results suggest that the age-related degradation of sound localization behavior might be caused by altered functions of higher stages in the auditory pathways above the level of the brainstem.

## Introduction

The ability to accurately locate the source of a sound, or sound localization, is a fundamental property of the auditory system, required for exploring the acoustic environment, performing effective communication, avoiding danger, and finding pray or mates [1,2]. Mammals, including humans, commonly possess specialized neuronal circuits for the encoding of binaural sound localization cues comprising minute differences in the sound waves arriving at the two ears. The lateral and medial superior olivary nuclei (LSO/MSO) in the auditory brainstem are among the first locations in the auditory system where the information from the two ears converges, with anatomical and synaptic properties suitable for extracting these small differences [1,2]. An LSO neuron receives excitatory inputs from the ipsilateral ear and inhibitory inputs originating from the contralateral ear [3,4]. Due to this binaural excitatory-inhibitory interaction, the output spike rate of an LSO neuron varies systematically with the interaural level difference (ILD), which is the disparity in the sound intensity levels between the two ears [5,6,7]. As a sound source is moved from one side of the head around to the other side, LSO neurons faithfully encode the corresponding ILD cues and therefore provide a neural correlate of sound location in azimuth [8,9]. Furthermore, in response to a periodic sound stimulus, LSO neurons act as an "anticoincidence detector" whose spike rate becomes maximal when excitatory and inhibitory synaptic inputs arrive temporally out-of-phase [10,11,12]. Because of this temporal processing property, LSO neurons are also sensitive to the binaural envelope phase difference of amplitude-modulated (AM) sounds, presenting periodic changes of spike rates locked to the envelope frequency [13].

**Fig 1:**
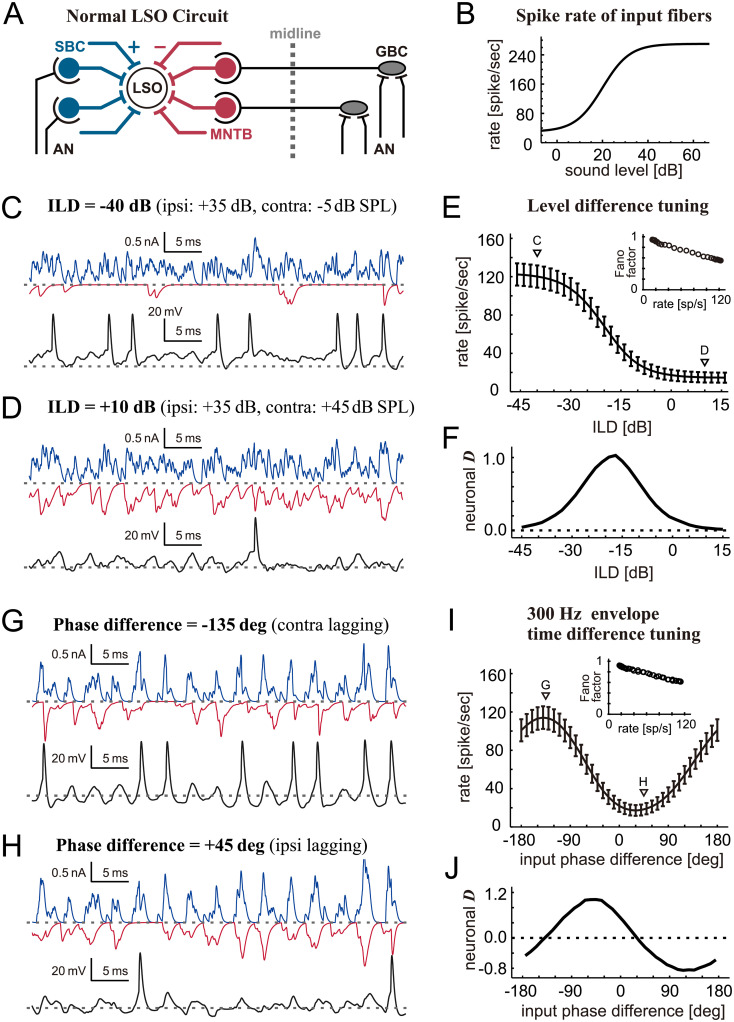
Modeled LSO functions. **A**. Schematic drawing of the LSO circuit. Excitatory inputs are shown in blue and back, and inhibitory inputs in red. AN: auditory nerve; GBC: globular bushy cell; LSO: lateral superior olive; MNTB: medial nucleus of the trapezoid body; SBC: spherical bushy cell. **B**. Modeled level-dependence of spike rates of SBCs and MNTB neurons used for simulating ILD coding. **C-D**. Simulated excitatory (blue) and inhibitory (red) synaptic inputs driven by binaural non-modulated tones with two different ILDs, and the resulting membrane potentials (black). Spike shapes are created by spike-mimicking current (see Materials and methods). **E**. Simulated ILD-tuning curve. Bars indicate the standard deviation of the spiking rate for 500-ms stimulations repeated 4000 times. Triangles show the ILD values used for the examples in C and D. (Inset) Fano factor of ILD-tuning curve. **F**. Neuronal discriminability for the ILD tuning curve (see Materials and methods for the definition). Neighboring ILD values in E (2-dB step) was used for the calculations. **G-H**. Simulated excitatory (blue) and inhibitory (red) synaptic inputs driven by binaural AM tones with two different input phase differences, and the resulting membrane potentials (black). **I**. Simulated binaural envelope phase-tuning curve at 300 Hz. Bars indicate the standard deviation of the spiking rate for each 500-ms stimulation. Triangles show the input phase differences used for the examples in G and H. (Inset) Fano factor of phase-tuning curve. **J**. Neuronal discriminability for the envelope phase-tuning curve. Neighboring phase difference values in I (10-deg step) was used for the calculations.

Behavioral studies have consistently showed that binaural hearing, including sound localization, generally degrades with age both in humans [14] (see [15,16] for reviews) and in model animals [17,18,19], despite these subjects having generally normal hearing detection thresholds (i.e., audibility of sound per se is not the cause of the decreased sound localization performance). The underlying neural mechanisms of age-dependent decay of auditory detection and discrimination, however, remain largely uncovered. One of the most salient changes found in studies of aged animals is the loss of inhibitory neurotransmission along the central auditory pathways [20,21]. In the sound localization circuit of the brainstem, inhibitory inputs from the medial nucleus of the trapezoid body (MNTB) to the LSO (Fig 1A) are selectively lost in aged rodents even without frank loss of hearing sensitivity (i.e., the animals typically have near normal hearing thresholds). In aged Sprague-Dawley rats, more than 30% of MNTB neurons are lost, and the decrease of inhibitory neurons starts already at 2–3 months of age [22]. Spongiform lesions appear in the MNTB of gerbils at ages much later than this (~3 years), indicating selective reduction of inhibitory neurons [23].

Degraded localization performance in aged animal models and humans might reasonably be hypothesized to result from the altered functions of the binaural brainstem circuits, including the LSO, caused by the loss of inhibitory inputs from MNTB. To date, however, there are only few empirical physiological studies of the aged LSO, and functional changes reported were rather subtle. In aged Sprague-Dawley rats, ILD-tuning curves were shifted toward the contralateral hemifield relative to younger animals, consistent with a reduction in inhibition, whereas other effects were not identifiable that were related to inhibition loss [24]. In Fisher-344 rats, loss of MNTB neurons assessed anatomically was less than 10% at 24 months relative to young animals [25]; and tuning-curves of LSO neurons were almost unaffected [26]. Studies of sound evoked auditory brainstem responses (ABR) have shown potentially reduced inhibitory efficacy at LSO, which has recently revealed to produce the binaural interaction component (BIC) of the ABR [27,28,29] (see [30] for review). The amplitude of the BIC in both elderly humans [31] and aged gerbils [32] is significantly reduced compared to younger subjects, despite relatively normal audibility. A reduction in the BIC amplitude is consistent with reduced efficacy of inhibition to the LSO [30,32]. These studies demonstrate that the anatomical reduction in inhibitory neurons can have functional consequences for LSO neurons, even if the changes noted are subtle.

In many areas of the nervous system, activity-dependent changes of the neuronal function, called homeostatic plasticity, play a fundamental role in facilitating, stabilizing and adjusting information processing [33,34,35]. A variety of adaptive plasticity takes place along the auditory system [36,37]. Activity-dependent alteration has also been found in the auditory brainstem, which was formerly regarded as "hard-wired" and not a target of plastic changes [38,39]. Noise-induced and age-related hearing loss trigger various functional and anatomical changes in the cochlear nuclei of rodents, but activity-dependent plasticity efficiently counteracts these changes to retain their fundamental functions [40]. These observations suggest the hypothesis that homeostatic plasticity may contribute to the preservation of binaural tuning in the aged LSO, despite the observed changes in the number and function of inhibitory neurons [4,20].

In the present study, we use a computational model of LSO [41] to examine the effects of age-related inhibition loss on the function of LSO. Our goals here are twofold. First, we study how the known reduction in the number of inhibitory inputs may or may not affect binaural tuning curves of LSO neurons. Second, we investigate how a compensatory increase of synaptic strength that counteracts the loss of inhibitory inputs may stabilize the binaural tuning of modeled LSO. With these analyses, we aim to gain insights on the relationship between known anatomical loss of inhibitory transmission in aged animals and the relatively unaffected physiological characteristics of this binaural nucleus.

## Materials and methods

### LSO neuron model

We used a single-compartment active integrate-and-fire (IF) model of LSO. The model was tuned to replicate known physiological response properties of LSO neurons [41]. The term "active" indicates that the model contains low-voltage-activated potassium (KLVA) conductance that plays a major role in auditory information processing [42,43]. We note that the limited amount of KLVA conductance in the LSO model does not alter its sustained firing pattern to current injections or its low-pass filtering property [41]. While anatomical [44,45] and physiological [46] studies reported multiple cell types in the LSO, we focus on neurons that show sustained spiking responses to constant acoustic stimulation, because the other major cell type, onset spiking neurons, are poor encoders of ILDs of continuous sounds.

The model equations and the corresponding parameter values are summarized in Table 1. The dynamics of the membrane potential *V* as well as the activation of the KLVA conductance are described each by a first-order differential equation. When the membrane potential reaches or exceeds the threshold *V*_θ_, an output spike is counted and the model is in an absolutely refractory period of *T*_ref_, in which no more spikes are counted. In addition, a spike-associated current *I*_spike_(*t -T*_θ_) is initiated at each threshold crossing to simulate a spike-like trajectory of the membrane potential.

**Table 1:** Equations and parameters for the active IF model. Units: *t* [ms], *V* [mV], *I*_spike_ [nA], *α*_*d*_ [1/ms], and *β*_*d*_ [1/ms]. Equations for the synaptic currents *I*_ex_ and *I*_inh_ are given in Table 3. See [41] for the complete description of this LSO neuron model and justification of the parameters.

**Cellular variable**	**Equation**
Membrane potential *V* (subthreshold dynamics)	$C\frac{d}{dt}V(t)=I_{\text{L}}+I_{\text{KL}}+I_{\text{ex}}+I_{\text{inh}}+I_{\text{spike}}$
Spike timing *T*_θ_	*T*_θ_: = *t* when *V*(*t*) ≥ *V*_θ_
Leak current	*I*_L_ = g_L_ (E_L_-*V*)
KLVA current	*I*_KL_ = g_KL_ *d*(*V*) (E_K_-*V*)
Spike-associated current (*s* = *t* -T_θ_ ≥0)	*I*_spike_(*s*) = 24exp(-s/0.15) − 12exp(-s/0.30)
KLVA activation variable *d*(t)	$\tau_{d}(V)\frac{d}{dt}d(t)=d_{\infty }(V)-d(t)$
Steady state for KLVA activation	*d*_∞_(*V*) = α_*d*_(*V*)/(α_*d*_(*V*) + β_*d*_(*V*))
Time constant for KLVA activation	τ_*d*_(*V*) = 1/(α_*d*_(*V*) + β_*d*_(*V*))
Activation rate of KLVA	α_*d*_(*V*) = 0.5exp(+(V+50)/16)
Deactivation rate of KLVA	β_*d*_(*V*) = 0.5exp(-(V+50)/16)
**Parameter**	**Value**
Membrane capacitance C	24 pF
Leak conductance g_L_	14.4 nS
KLVA conductance g_KL_	21.6 nS
Leak reversal potential E_L_	-56 mV
Potassium reversal potential E_K_	-75 mV
Threshold *V*_θ_	-45.8 mV
Refractory period *T*_ref_	1.6 ms

In addition to the active IF model, we also used a passive IF model to confirm the generalizability of our results. The parameters of this model (Table 2) were taken from a previous study [41], in which a general comparison between the active and the passive model was performed. The passive IF model lacks any active voltage-dependent conductance. When its membrane potential *V* reaches the threshold *V*_θ_, a spike is counted and then the membrane potential *V* is fixed to the reset potential *V*_0_ for a refractory period of *T*_ref_. The passive IF model was used only in the specific subsection titled "Binaural tuning simulated with passive IF model" in the Results. In all other sections, we used the active IF model described above.

**Table 2:** Equations and parameters for the passive IF model. See [41] for the complete description of this LSO neuron model and justification of the parameters.

Parameter	Value
Membrane capacitance C	24 pF
Leak conductance g_L_	26.4 nS
KLVA conductance g_KL_	0 nS
Leak reversal potential E_L_	-60 mV
Reset potential *V*_0_	-60 mV
Threshold *V*_θ_	-45.1 mV
Refractory period *T*_ref_	1.6 ms

### LSO input model

We adopted the same configurations for the excitatory and inhibitory synaptic inputs to the modeled LSO neuron as in our previous study [41]. The model equations and the corresponding parameter values are summarized in Table 3. Each presynaptic spike is converted into a unitary synaptic conductance modeled as an alpha function (*α*_ex_ or *α*_inh_), and is then summed into the total excitatory (g_ex_) and inhibitory (g_inh_) synaptic conductances. The spiking activity of the excitatory and inhibitory input fibers (from spherical bushy cells and MNTB neurons, respectively; see Fig 1A) were modeled as a Poisson process. For non-modulated tones used for simulating ILD-tuning curves, the Poisson process is homogeneous in time with a level-dependent intensity function λ(SPL) (Fig 1B). For AM tones used for simulating phase-tuning curves, the Poisson process is inhomogeneous, with a von Mises distribution function used as the underlying intensity function [47]. In this case, the spiking activity is characterized by two parameters, the average intensity λ(*f*_*m*_) and the degree of phase-locking VS(*f*_*m*_), with *f*_*m*_ being the modulation frequency. We assumed that all the inputs are mutually independent and that, in phase-locked cases, all excitatory inputs are locked to one single phase and the inhibitory inputs are locked to another. See [47] for the detailed theoretical relationship between the parameters of the von Mises distribution and the degree of phase-locking measure as the vector strength (VS) [48].

**Table 3:** Equations and parameters for excitatory and inhibitory synaptic inputs. *I*_ex_^*m*^ and *I*_inh_^*m*^ are the number of spikes of the *m*-th excitatory and inhibitory fibers, respectively. *t*^*m*^_*i*_ denotes the timing of the *i*-th spike of the *m*-th fiber. *f*_*m*_ is the modulation frequency in Hz. SPL is the sound level in dB. The unit for the intensities λ is spikes/s. See [41] for the complete description of the synapse model and justification of the parameters.

**Synaptic variable**	**Equation**
Unitary excitatory synaptic conductance	α_ex_(*t*) = *A*_ex_(*t*/τ_ex_)exp(1-t/τ_ex_) (*t* ≥ 0)
Total excitatory synaptic conductance	$g_{\text{ex}}(t)=\sum_{m=1}^{M_{\text{ex}}}\sum_{i=1}^{I_{\text{ex}}^{m}}\alpha_{\text{ex}}(t-t_{i}^{m})$
Excitatory synaptic current	*I*_ex_(*t*) = g_ex_(*t*) (E_ex_-*V*)
Unitary inhibitory synaptic conductance	α_inh_(*t*) = *A*_inh_(*t*/τ_inh_)exp(1-t/τ_inh_) (*t* ≥ 0)
Total inhibitory synaptic conductance	$g_{\text{inh}}(t)=\sum_{m=1}^{M_{\text{inh}}}\sum_{i=1}^{I_{\text{inh}}^{m}}\alpha_{\text{inh}}(t-t_{i}^{m})$
Inhibitory synaptic current	*I*_inh_(*t*) = g_inh_(*t*) (E_inh_-*V*)
**Parameter**	**Value**
Peak amplitude of excitatory input conductance A_ex_	3.5 nS
Peak amplitude of inhibitory input conductance A_inh_	12 nS (default)
Time constant of excitatory input conductance τ_ex_	0.16 ms
Time constant of inhibitory input conductance τ_inh_	0.32 ms
Reversal potential for excitatory inputs E_ex_	0 mV
Reversal potential for inhibitory inputs E_inh_	-75 mV
Number of excitatory input fibers M_ex_	20
Number of inhibitory input fibers M_inh_	8 (default)
**Input variable**	**Equation**
Level-dependent intensity function for non-modulated tones	$\lambda (\text{SPL})=30+\frac{240}{1+\text{exp}(-(\text{SPL}-20)/6.0)}$
Modulation-frequency-dependent average intensity	λ(*f*_m_) = 180 − 0.03*f*_*m*_
Modulation-frequency-dependent phase-locking (*f*_*m*_<2000)	$\text{VS}(f_{m})=0.65\times \frac{1-\text{exp}((f_{m}-2000)/500)}{1+\text{exp}((f_{m}-2000)/500)}$

### Testing level- and phase-tuning

ILD-tuning of the LSO model for non-modulated tones was simulated by varying the contralateral sound intensity level that determines the inhibitory synaptic input intensity (Table 3). The ipsilateral sound pressure level was fixed to +35 dB. As in most physiological studies (e.g., [6,7,46]), the ILD was defined as the contralateral level minus ipsilateral level. ILD sensitivity of binaural neurons throughout the auditory system, from LSO to cortex, does not depend on the frequency sensitivity (i.e., characteristic frequency) of the neuron [49]. Thus, the model LSO neuron is representative of typical LSO neurons regardless of their frequency sensitivity.

Envelope phase-tuning of the LSO model for AM tones was simulated by varying the relative phase between the ipsilateral (excitatory) and contralateral (inhibitory) inputs. Namely, the difference between the locking phases of both inputs was varied from -180 to +180 degrees. A positive phase means that inhibitory inputs arrive earlier than excitatory inputs. Unless otherwise noted, the modulation frequency was fixed to 300 Hz, at which the effect of amplitude-modulation is highly prominent [11].

Tuning curves of the model LSO neuron were further characterized with two measures: modulation depth and neuronal discriminability. The modulation depth is defined as the difference between the maximum and minimum spiking rate of the tuning curve. The neuronal discriminability *D* is defined as the spike rate difference between two stimulus conditions normalized by the mean standard deviation of the spike rates [50]: $D=({\text{μ}}_{1}-{\text{μ}}_{2})/\sqrt{(\sigma_{1}^{2}+\sigma_{2}^{2})/2}$. Here the suffixes 1 or 2 denote two different stimulus values of ILD or input phase difference, and *μ* and *σ* are the mean and standard deviation of the spike rate for each stimulus value, respectively. The discriminability can be theoretically related to the Fisher information *I*_F_(*S*) as: $|D|=\Delta S\sqrt{I_{\text{F}}(S)}$, where *S* denotes the stimulus value and Δ*S* is the difference between two neighboring stimulus values (see section 3.3 of [51]). In other words, the discriminability is a statistical measure that indicates how precisely a downstream neuron can theoretically infer the value of the input parameter (i.e., ILD or phase difference) by observing the spike rate of the upstream neuron (i.e., LSO). In the present study, the step size was Δ*S* = 2 dB for an ILD-tuning curve and Δ*S* = 10 degrees for a phase-tuning curve. For each ILD or phase value, we repeated 500-ms sound stimulation by 4000 times to calculate the mean and standard deviation of the output spike rates of our LSO neuron model.

The Fano factor, which is the spike count variance in a fixed period divided by the average number of spikes, is often used to characterize the variation of spiking response. LSO neurons typically have a Fano factor near or slightly below 1, indicating somewhat more regular spiking activity than the Poisson process [52]. Our simulated spike variance matched corresponding empirical observations, including the decrease of the Fano factor with the spike rate (insets of Fig 1E and 1I).

### Loss of inhibitory inputs and amplitude compensation

Age-related loss of inhibitory neurons was simulated by reducing the number of inhibitory synaptic inputs. In the default (healthy) condition, the number M_inh_ of inhibitory input to the model LSO neuron was fixed to 8 (Table 3), which is based on available experimental data in gerbils [53] and mice [54]. We varied this parameter between 0 and 16, and calculated the binaural level and phase tuning curves of the model LSO neuron.

In order to examine the effects of inhibition loss, we considered three cases named "uncompensated", "compensated", and "overcompensated". In the uncompensated case, only the number of inhibitory inputs was changed, while all the other parameters were kept unchanged. In the compensated case, the unitary amplitude magnitude of inhibitory conductance was varied according to the number of remaining inhibitory inputs so that the total amount of inhibitory conductance remained unchanged. In this second case, when the number of inhibitory inputs was halved from M_inh_ = 8 to 4, for example, the unitary amplitude was doubled from A_inh_ = 12 to 24 nS to compensate the loss. In the overcompensated case, the amplitude of the unitary inhibitory input was multiplied by a factor (2—M_inh_/8), so that the total inhibitory input conductance linearly decreased with the number of inputs. For instance, the total inhibitory input became 50% larger than the default value when the number of inputs was 4. Even in these compensated and overcompensate cases, all model parameters other than the number and amplitude of inhibitory inputs were unchanged throughout the simulations.

### Population modeling

While the spike rate of an LSO neuron monotonically decreases with ILD (e.g., Fig 1E), the location of the midpoint of an ILD-tuning curve generally differs between neurons [55,56]. To investigate how loss of inhibitory inputs may affect neuronal discriminability function on the population level, we created a set of LSO neurons by shifting the tuning curves along the ILD axis. The resulting LSO neuron population had tuning curves whose midpoint locations uniformly distributed between -26 and +16 dB, covering more than 80% of the empirical distribution in gerbil LSO [56].

For each of the LSO neurons, we next assumed a "mirrored neuron" whose ILD-tuning function is a flipped image of the original tuning curve about the midline (zero ILD). This mirrored neuron is the delegate of a similarly-tuned neuron in the contralateral LSO. We then calculated the spike rate difference between the original and mirrored neuron to obtain the bilateral rate difference (similar approaches were employed in [7,49,57,58]). This rate difference is assumed to carry the ILD information required for horizontal sound localization [7,58]. Applying the same formula as described above, we computed the neuronal discriminability function from the across-trial mean and standard deviation of the bilateral rate difference.

As there is no empirical data available on how outputs of multiple LSO neurons are integrated at higher auditory stages, we calculated the "population discriminability" by simply averaging the neuronal discriminability functions across the LSO population. The underlying assumptions here are that sufficiently many neurons with different midpoint locations are involved and that each neuron in the population shares the same impact on the resulting discriminability through averaging. The discriminability functions were calculated for different numbers of inhibitory inputs in the uncompensated and compensated conditions. For this population averaging, we adopted 22 mirror neuron pairs in total (having midpoint locations from -26 to +16 dB with a step of 2 dB). We note that the number of neuron pairs used would not affect our conclusion, since the calculated discriminability was all normalized to eliminate the influence of the population size.

## Results

### Simulating binaural tuning in healthy LSO

LSO neurons change their spike rates according to binaural sound level differences and to binaural envelope phase differences. As demonstrated in our previous study [41], these fundamental characteristics can be replicated with our LSO model (Fig 1). When the ipsilateral sound level is much higher than the contralateral sound level, excitatory inputs to the model LSO cell are much stronger than inhibitory inputs, therefore leading to a high spike rate (Fig 1C). For more intense contralateral sound stimulation, simulated inhibitory inputs more effectively cancel excitatory inputs, resulting in a lower spike rate (Fig 1D). Because of this excitatory-inhibitory interaction, the level difference tuning curve of an LSO neuron generally has a sigmoidal shape (Fig 1E). And the neuronal discriminability becomes maximal at or near the ILD where the slope of the binaural tuning function is steepest (Fig 1F). As the LSO model itself does not have an internal noise source, the observed trial-to-trial variability in its output spike rate is caused solely by the randomness of the synaptic inputs generated with the Poisson process.

In response to binaural AM sounds, the model LSO generates many spikes when phase-locked excitatory inputs precede inhibitory inputs (Fig 1G). In such a case, the oscillation of the membrane potential is large and therefore the spike threshold is reached easily. In contrast, when the inhibitory inputs arrive slightly earlier than excitatory inputs, the peaks of both inputs coincide and cancel each other, leading to a low spike rate (Fig 1H). If the inhibitory inputs further precede the excitatory inputs, then the spike rate again increases. Because of the periodic nature of this phase-dependent binaural interaction, the resulting phase-difference tuning curve is sinusoidal (Fig 1I). The neuronal discriminability function is also periodic (Fig 1J), with positive and negative maximal values located close to the positions that correspond to the steepest slopes of the tuning curve (Fig 1I).

### Simulating effects of inhibition loss on binaural tuning

**Fig 2:**
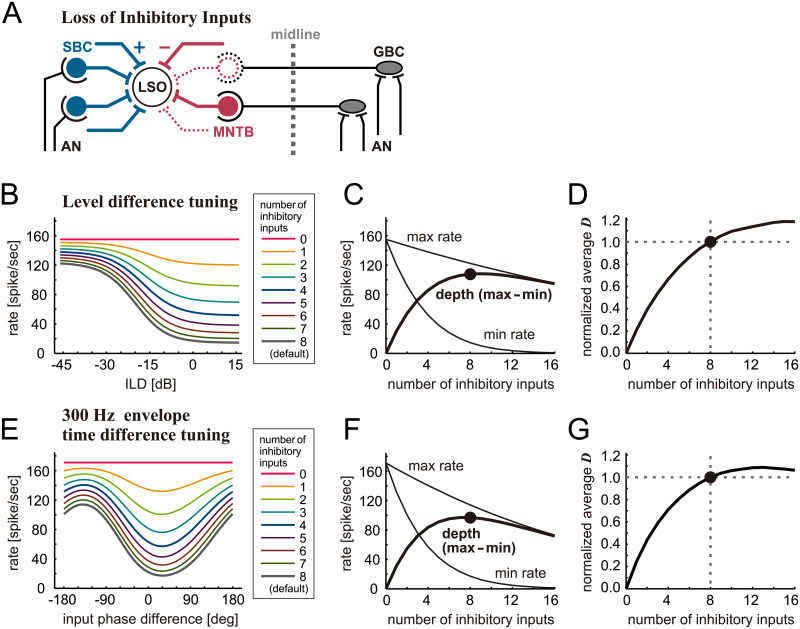
Simulated effects of inhibition loss on binaural tuning of LSO without amplitude compensation. **A**. Schematic drawing of the LSO circuit with a loss of inhibitory synaptic inputs. Lost inputs are shown by dotted lines. **B**. Simulated ILD-tuning curves for different numbers of inhibitory inputs. **C**. (Thin lines) peak (max) and trough (min) rates of the ILD-tuning curves. (Thick line) Modulation depth defined as the difference between maximum and minimum rates. **D**. Neuronal discriminability of ILD tuning curves averaged over the range between -45 and +15 dB and normalized to the value for 8 inhibitory inputs. **E**. Simulated envelope phase-tuning curves at 300 Hz for different numbers of inhibitory inputs. **F**. (Thin lines) peak (max) and trough (min) rates of the envelope phase-tuning curves. (Thick line) Modulation depth defined as the difference between maximum and minimum rates. **G**. Neuronal discriminability of envelope phase-tuning curves averaged over the range between -180 and +180 degrees and normalized to the value for 8 inhibitory inputs. In panels **C**, **D**, **F**, and **G**, the filled circles show the response for the default number of inputs (M_inh_ = 8).

To simulate age-related loss of inhibition, we changed the number of inhibitory inputs to the model LSO and examined how binaural level- and phase-tuning curves are altered. First, we considered an "uncompensated" case, in which only the number of inhibitory inputs was varied from the default value of 8, while all other model parameters remained unchanged (Fig 2A). This situation would correspond to an acute loss of inhibitory inputs without any recovery processes. In our series of simulations, the number of inputs was varied between 0 and 16 for completeness. Considering the number of inputs in young LSO [53,54] and the amount of its reduction by aging [22,25], however, we assumed that the most realistic range for the number of inhibitory inputs to an LSO neuron would be 4–12, on which we primarily focus in the following subsections.

Simulated ILD-tuning curves showed a gradual increase of spike rates with a decreasing number of inhibitory inputs and became flat when all the inhibitory inputs were lost (Fig 2B). Midpoints of the sigmoidal tuning curves slightly shifted to positive ILDs for a smaller number of inhibitory inputs (e.g., from -20.0 dB for M_inh_ = 8 inputs to -17.3 dB for M_inh_ = 4 inputs). This shift of midpoints qualitatively agrees with previous experimental findings [24]. The maximum rate (peak of the tuning curve) changed almost linearly with the number of inhibitory inputs, while the minimum rate (trough of the tuning curve) changed non-linearly, presumably because the spike rate cannot be below zero (Fig 2C, thin lines). The resulting modulation depths increased almost linearly for 0–4 inputs, saturated for 6–10 inputs, and slowly decreased for 12–16 inputs (Fig 2C). The neuronal discriminability *D* was averaged over the entire ILD range tested and normalized to its value for M_inh_ = 8 inputs. The curve of this normalized discriminability monotonically increased with the number of inhibitory inputs and saturated (Fig 2D). The reduction of discriminability with inhibition loss is caused by two factors: reduced spike rate differences (Fig 2B) and increased rate variance associated with the increased average spike rate (Fig 1E). For M_inh_ = 6 inputs and above, the discriminability differed, however, by less than 15% from the default case of M_inh_ = 8 inputs.

Simulated envelope phase-tuning curves also showed gradual changes with the number of inhibitory inputs (Fig 2E). Fewer inhibitory inputs led to higher overall spike rates with shallower modulation depths. As in level-tuning, the maximum rates decreased linearly with the number of inhibitory inputs, while the minimum rates changed nonlinearly (Fig 2F, thin lines). The resulting modulation depth had a peak at around 6–8 inputs (Fig 2F, thick line). The neuronal discriminability *D* increased with the number of inhibitory inputs and had a mild peak at around M_inh_ = 12 inputs (Fig 2G). These results indicate that binaural level and phase difference tunings (as measured by the modulation depths or by discriminability) are not strongly affected by the exact number in the range of 6–12 inhibitory inputs.

### Simulating amplitude compensation acting against inhibition loss

**Fig 3:**
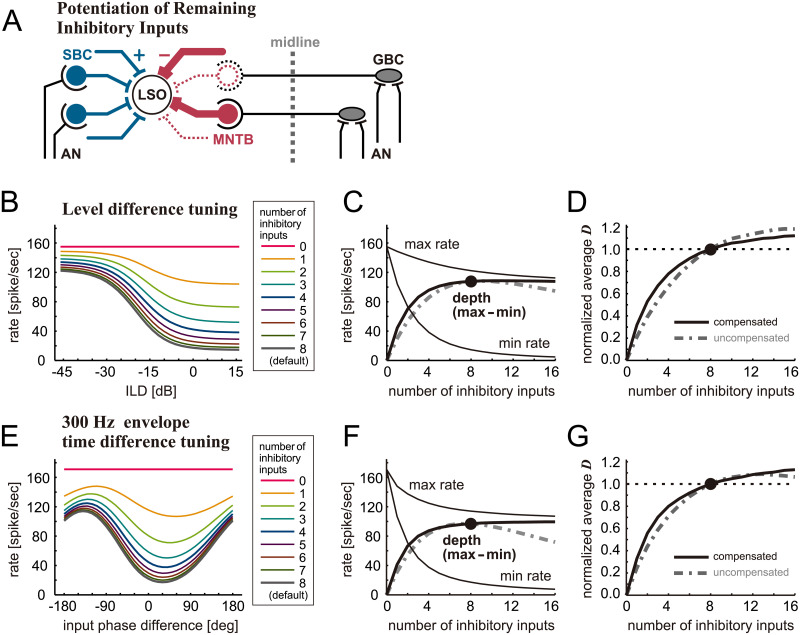
Simulated effects of inhibition loss on binaural tuning of LSO with amplitude compensation. **A**. Schematic drawing of the LSO circuit with a loss of inhibitory synaptic inputs and potentiation of remaining inputs. Lost inputs are shown by dotted lines and potentiated inputs are indicated by thick lines. **B**. Simulated ILD-tuning curves for different numbers of inhibitory inputs. **C**. (Thin lines) peak (max) and trough (min) rates of the ILD-tuning curves. (Thick solid line) Modulation depth defined as the difference between maximum and minimum rates. **D**. Neuronal discriminability of ILD tuning curves averaged over the range between -45 and +15 dB and normalized to the value for 8 inhibitory inputs. **E**. Simulated envelope phase-tuning curves at 300 Hz for different numbers of inhibitory inputs. **F**. (Thin lines) peak (max) and trough (min) rates of the envelope phase-tuning curves. (Thick solid line) Modulation depth defined as the difference between maximum and minimum rates. **G**. Neuronal discriminability of envelope phase-tuning curves averaged over the range between -180 and +180 degrees and normalized to the value for 8 inhibitory inputs. In panels **C**, **D**, **F**, and **G**, simulated data for the uncompensated case (same curves as in the corresponding panels in Fig 2) are shown by gray dot-dashed lines for comparison; the filled circles show the response for the default number of inputs (M_inh_ = 8).

Next, we considered a "compensated" case in which the magnitude of remaining inhibitory inputs was increased to counteract the reduction in the number of inhibitory inputs (Fig 3A; see Materials and methods for more details). This case might correspond to the situation where homeostatic and other kinds of neuronal plasticity take place to partly recover from the loss of synaptic inputs. Here, the average spike rate, which was increased due to the loss of inhibition, was then reduced by augmenting the impact of remaining inhibitory synapses.

In comparison to the uncompensated case (shown in Fig 2), effects of inhibition loss were greatly reduced, when the loss of inhibition was compensated by amplifying the remaining inhibitory inputs. Simulated ILD-tuning curves became substantially shallow only when more than half of the inhibitory inputs were lost (Fig 3B). This limited effect can also be seen by plotting the modulation depth (Fig 3C, thick solid line). Unlike the uncompensated case, both maximum and minimum rates showed nonlinear changes with the number of inhibitory inputs in the compensated case (Fig 3C, thin lines). More specifically, the changes of these rates were drastic for small numbers of inputs (M_inh_ = 0–3) and only marginal for larger numbers of inputs (M_inh_ ≥ 4). As a result, the simulated modulation depth showed a prominent change for 0–4 inputs but was almost constant for the range above 4 inputs (Fig 3C, thick line). A similar trend was found for the normalized neuronal discriminability (Fig 3D). Around the default number of inhibitory inputs (M_inh_ = 8), the curves for the modulation depth and the discriminability in the compensated case were even shallower than in the uncompensated case (gray dash-dotted lines). These observations indicate that loss of a small to moderate number of inhibitory fibers does not impair binaural tuning in LSO.

Simulated envelope phase-tuning showed comparable or even stronger robustness against the loss of inhibition (Fig 3E). The shape of the phase-tuning curves was affected only slightly even when half of the inhibitory inputs were lost (compare 4 and 8 inputs in Fig 3E). This observation is confirmed with the modulation depths plotted against the number of inhibitory inputs (Fig 3F). Amplitude compensation contributed to a flattening of the modulation depth over 4 inhibitory inputs. Further compensatory effects are demonstrated for the normalized discriminability *D* (Fig 3G), whose amount of decrease was less than 10% for M_inh_ ≥ 6 inputs and less than 25% for M_inh_ ≥ 4 inputs. To sum, when the amplitude compensation mechanisms take place, the binaural tuning curves becomes even more robust to changes of the number of inhibitory inputs.

### Simulating amplitude overcompensation

**Fig 4:**
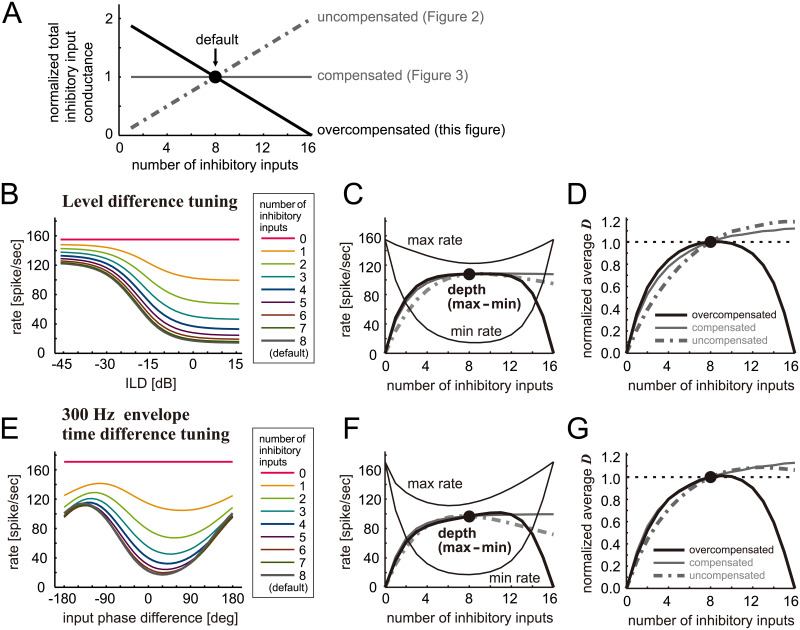
Simulated effects of inhibition loss on binaural tuning of LSO with amplitude overcompensation. **A**. Total amount of inhibitory synaptic inputs normalized to the value for the default (M_inh_ = 8 inputs). In the uncompensated case (gray dot-dashed line; data shown in Fig 2), the total inputs depended linearly on the number of inputs, while in the compensated case (thin gray line; data shown in Fig 3), the total inputs were kept constant. In the present overcompensation case (solid black line), the summed inhibitory conductance was a linear decreasing function of the number of inhibitory inputs. Namely, in comparison to the default condition, the LSO neuron model receives stronger inhibition in total, when the number of inhibitory inputs was reduced. **B**. Simulated ILD-tuning curves for different numbers of inhibitory inputs. **C**. (Thin lines) peak (max) and trough (min) rates of the ILD-tuning curves. (Thick solid line) Modulation depth defined as the difference between maximum and minimum rates. **D**. Neuronal discriminability of ILD tuning curves averaged over the range between -45 and +15 dB and normalized to the value for 8 inhibitory inputs. **E**. Simulated envelope phase-tuning curves at 300 Hz for different numbers of inhibitory inputs. **F**. (Thin lines) peak (max) and trough (min) rates of the envelope phase-tuning curves. (Thick solid line) Modulation depth defined as the difference between maximum and minimum rates. **G**. Neuronal discriminability of envelope phase-tuning curves averaged over the range between -180 and +180 degrees and normalized to the value for 8 inhibitory inputs. In panels **C**, **D**, **F**, and **G**, simulated results for the uncompensated case (gray dot-dashed lines; same data as in the corresponding panels in Fig 2) and for the perfectly compensated case (thin gray lines; same data as in the corresponding panels in Fig 3) are shown for comparison; the filled circles show the response for the default number of inputs (M_inh_ = 8).

In the above compensated case, we considered an ideal situation where the total inhibitory conductance remained unchanged from the default, regardless of how many inhibitory synapses were lost. In a real biological system, however, compensatory mechanisms should be more dynamically regulated, and improper compensation can lead to erroneous percepts [59]. In the auditory system, maladaptive neuronal hyperactivity is suggested to cause tinnitus [60]. In this subsection, we consider an "overcompensated" case in which the amplification of remaining inhibitory inputs to the LSO model exceeded the total loss of inhibitory conductance (Fig 4A). The aim of this hypothetical setting is to examine whether the compensation mechanism needs to be precisely controlled to achieve stable binaural tuning.

The simulated tuning curves in the overcompensated case (Fig 4B and 4E) resembled those for the compensated case (Fig 3B and 3E). This similarity is confirmed by the modulation depth plots (Fig 4C and 4F). For 4–12 inhibitory inputs, the dependence of the depth of level and phase tuning curves on the number of inhibitory inputs was nearly indistinguishable between these compensatory conditions (solid black and solid gray lines). For M_inh_ = 16 inputs, however, the modulation depth vanishes in the overcompensation case, because of the input amplitude equals to zero, which can be considered unphysiological (Fig 4A). The normalized discriminability curve for ILD-tuning in the overcompensated case was even shallower than in the compensated case (Fig 4D), suggesting that additional increase of the remaining inhibition may further contribute to the stability of ILD tuning. In binaural phase-tuning, however, the normalized discriminability functions for the compensated and overcompensated cases showed very similar trajectories for 3–9 inputs, implying minimal effects of overcompensation (Fig 4G).

Our simulation results indicate that the fraction of performance reduction is generally smaller than the amount of inhibition loss, and this reduction in performance is further compressed by amplitude compensation. In one measure (modulation depth for phase difference), however, (over)compensation did not improve the performance, because the overall rate increase in the uncompensated case counteracted the decrease of the modulation depth. In sum, our simulation results suggest that activity-dependent plasticity may effectively counteract the age-dependent loss of inhibitory fibers projecting to the LSO, and that this robustness of binaural tuning does not strongly depend on the exact amount of compensation.

### Phase-tuning at different modulation frequencies

**Fig 5:**
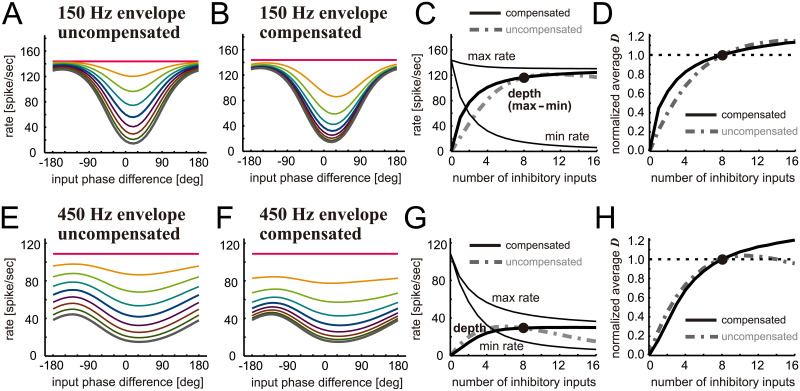
Simulated effects of inhibition loss on binaural phase tuning of LSO. **A-D**. Responses of the model LSO neuron to 150-Hz amplitude-modulated sounds. **E-H**. Responses of the model LSO neuron to 450-Hz amplitude-modulated sounds. Panels **A, B, E** and **F** present simulated envelope phase-tuning curves for different numbers of inhibitory inputs either without (**A,E**) or with (**B,F**) amplitude compensation. The color code used is the same as in Figs 2–4. Thin lines in **C** and **G** show peak (max) and trough (min) rates of the corresponding phase-tuning curves in **B** and **F**, respectively. Thick solid lines in **C** and **G** show the modulation depth defined as the difference between maximum and minimum rates for the compensated case, while gray dot-dashed lines show that for the uncompensated case. **D** and **H** present the neuronal discriminability of envelope phase-tuning curves averaged over the range between -180 and +180 degrees and normalized to the value for 8 inhibitory inputs either for the compensated case (solid black) or uncompensated case (dot-dashed gray).

Our results in the previous subsections suggest that binaural tuning in LSO is robust against changes in the number of inhibitory inputs. In the remaining part of Results, we confirm this conclusion at different simulation settings. In this subsection, we examine the phase-tuning of the model LSO at two additional modulation frequencies of 150 and 450 Hz (Fig 5). As observed in previous in vivo measurements [13,61], the trough positions of simulated phase-tuning curves coincide across different modulation frequencies, while the shapes of tuning curves may vary (Figs 2E, 5A and 5E). At 150 Hz, the number of inhibitory inputs affects the trough spiking rate most strongly (Fig 5A). This effect, however, is reduced by the amplitude compensation (Fig 5B), resulting in a curve of modulation depth that is almost flat around M_inh_ = 8 inputs (Fig 5C). The normalized discriminability for 150 Hz (Fig 5D) showed a very similar dependence on the number of inputs as for 300 Hz (Fig 3G).

Phase-tuning curves at 450 Hz (Fig 5E) were shallower than those at 150 or 300 Hz, because of the combination of weaker phase-locking of input fibers and shorter modulation period. Such shallower tuning curves were observed both in physiological measurements [13,61] and in computer simulations [11,41]. At 450 Hz, the number of inhibitory inputs affected not only the trough rate but also the overall excitability of the model (Fig 5E). Namely, the tuning curve became higher and narrower as the number of inhibitory inputs decreased (Fig 5G). Amplitude compensation counteracted these changes (Fig 5F), but overall its contribution was subtle at this high modulation frequency (Fig 5G and 5H). Unlike the example of 150 Hz, the overall spike rate greatly rises with decreased inhibition, which partly counteracts the reduction of modulation depth even for the uncompensated case (Fig 5A and 5D). Because of this effect, the difference between the compensated and the uncompensated cases becomes minimal at 450 Hz. To sum, phase-tuning in LSO is robust against inhibition loss at all modulation frequencies tested (150, 300 and 450 Hz), while the effect of amplitude compensation may be small at high modulation frequencies.

### Binaural tuning simulated with passive IF model

Up to the last subsection, we have used an active IF model, which has a small amount of voltage-dependent KLVA conductance. This conductance slightly reduces the maximum spiking rate for an ILD-tuning curve by suppressing the accumulating inputs (e.g., [43]), and slightly enhances the peak of a phase-tuning curve probably through post-inhibitory facilitation [12], making the model better fit corresponding experimental data than a model without KLVA conductance [41]. However, the amount of KLVA in the LSO model is only limited, especially compared to MSO (e.g., [62]), such that the modeled neuron still shows a low-pass filtering property to subthreshold inputs and tonic spiking to large suprathreshold inputs [41].

**Fig 6:**
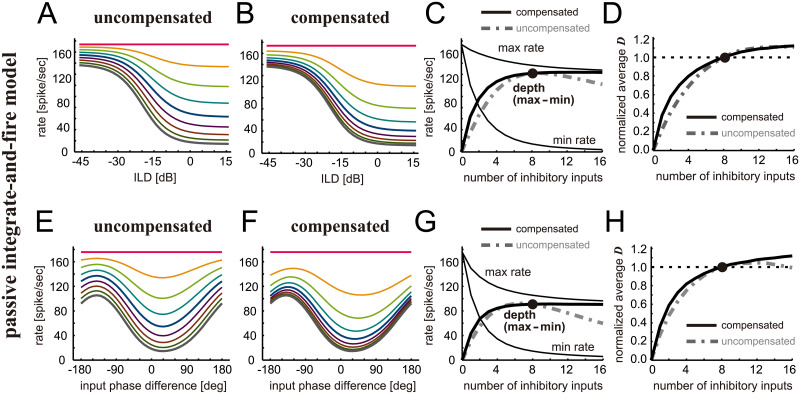
Simulated effects of inhibition loss on binaural tuning of LSO modeled with a passive IF model. **A-D**. Simulated ILD-tuning. **E-H**. Simulated envelope phase-tuning at 300 Hz. Panels **A, B, E** and **F** present simulated binaural ILD- or phase-tuning curves for different numbers of inhibitory inputs either without (**A,E**) or with (**B,F**) amplitude compensation. The color code used is the same as in Figs 2–4. Thin lines in **C** and **G** show peak (max) and trough (min) rates of the corresponding binaural tuning curves in **B** and **F**, respectively. Thick solid lines in **C** and **G** show the modulation depth defined as the difference between maximum and minimum rates for the compensated case, while gray dot-dashed lines show that for the uncompensated case. Panel **D** presents neuronal discriminability of ILD tuning curves averaged over the range between -45 and +15 dB and normalized to the value for 8 inhibitory inputs. Panel **H** presents the neuronal discriminability of binaural tuning curves averaged over the range between -180 and +180 degrees and normalized to the value for 8 inhibitory inputs. In **D** and **H**, solid black lines indicate the compensated case, while gray dash-dotted lines indicate the uncompensated case.

To test if the existence of this KLVA conductance affects our conclusion of the robustness of binaural tuning in LSO, we repeated the same simulations using a passive IF model without any active conductances (see Materials and methods). Despite some difference in overall spiking rates, both active IF (Fig 2B and 2E) and passive IF (Fig 6A and 6E) model showed very similar dependence of binaural tuning curves on the number of inhibitory inputs. The effects of amplitude compensation were also similar between the two models (Figs 3B, 3E, 6B and 6F). With amplitude compensation, the resulting modulation depths (Fig 6C and 6G) and neuronal discriminability (Fig 6D and 6H) were almost unchanged for 6–12 inhibitory inputs in the passive IF model, closely resembling our observation for the active IF model (Fig 3C, 3D, 3F and 3G). These comparisons indicate that adding some KLVA conductance to make the modeled outcome realistic does not affect the robustness of binaural tuning against the loss of inhibition.

### Simulating population coding

**Fig 7:**
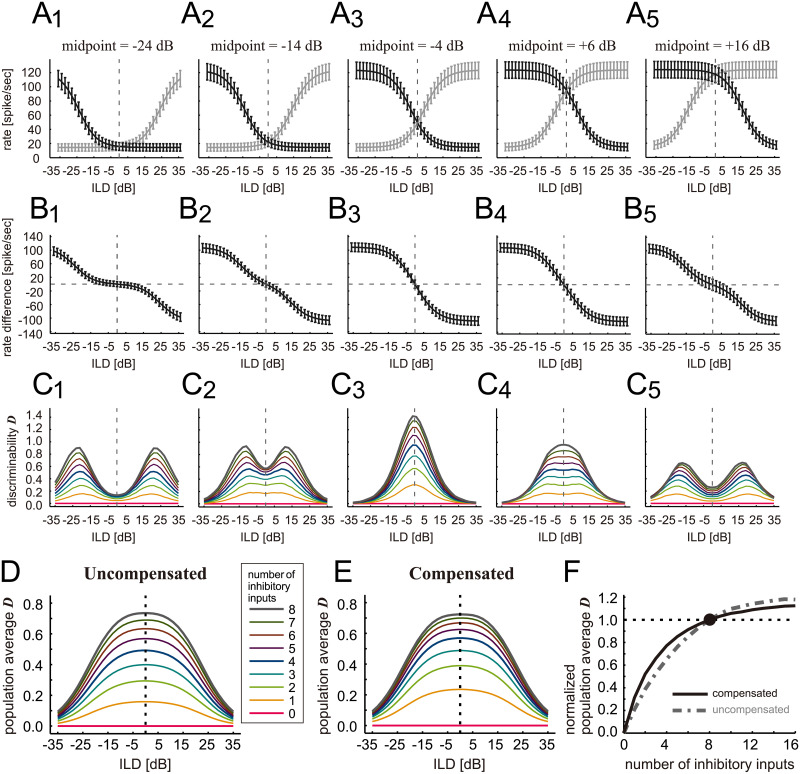
Simulated effect of inhibition loss on LSO population coding. **A1-A5**. Simulated ILD-tuning curves with varied midpoint locations (black) and their mirrored images (gray). Bars indicate the standard deviation of the spiking rate for repeated 500-ms stimulations. **B1-B5**. Bilateral spike rate differences, defined as the rate difference of each LSO pair (i.e., original and mirrored). **C1-C5**. Neuronal discriminability functions calculated from the bilateral spike rate differences. **D**. Population average of the neuronal discriminability functions, plotted for different number of inhibitory inputs without amplitude compensation. **E**. Population average of the neuronal discriminability functions, plotted for different number of inhibitory inputs with amplitude compensation. **F**. Population-averaged discriminability that was further averaged over the ILD range between -35 and +35 dB and normalized to the value for 8 inhibitory inputs.

In previous subsections, we have investigated possible effects of inhibition loss on binaural tuning at the single cell level. To test our results also at the population level, we simulated a set of LSO neurons whose tuning curves had the steepest slope at different ILDs (Fig 7A1–7A5, black curves), matching the empirical curve distribution in gerbils [56]. For each LSO neuron, we introduced a mirrored neuron [7,49,57,58] that would encode the ILD of sound sources located contralaterally to the original LSO (Fig 7A1–7A5, grey curves). We next calculated the binaural spike rate difference between the original and the mirrored neuron (Fig 7B1–7B5). From this rate difference, we then computed the neuronal discriminability *D* of each LSO pair (Fig 7C1–7C5). When the original ILD-tuning curve is steepest near zero ILD, the resulting discriminability function becomes unimodal (panels C3 and C4). In contrast, when the dynamic ranges of the two mirrored ILD-tuning curves do not overlap, the discriminability function is bimodal (panels C1, C2, and C5) [49]. In both cases, a loss of inhibitory inputs generally reduces the discriminability for all LSO pairs, while the overall shape of each curve is roughly preserved (Fig 7C1–7C5, colored lines).

By averaging the discriminability functions across neuron pairs with different midpoint locations, we obtained the population-averaged discriminability for our set of LSO neurons. The curve for the population-averaged discriminability was unimodal but notably flat in the range of ±10 dB (Fig 7D), because of the averaging of unimodal and bimodal curves whose peaks are located at different positions. The high discriminability around zero ILD seems to correspond to the good sound localization precision near the midline reported in behavioral studies (e.g., [63]). We calculated the population-averaged discriminability with reduced numbers of inhibitory inputs with and without amplitude compensation (Fig 7D and 7E). In both cases, loss of inhibition reduces the discriminability without altering the shape of the curves. To examine the effect of amplitude compensation, we further averaged the population discriminability function in the range of ±35 dB and normalized it to the value for the default condition of 8 inhibitory inputs (Fig 7F). The resulting curve for the normalized population discriminability closely resembled the discriminability function of a single neuron (Fig 3D), confirming the conclusions that the simulated ILD-tuning functions are generally robust to a loss of inhibition and that robustness might be further boosted by amplitude compensation.

## Discussion

### Simulating age-related loss of inhibition in LSO

Age-related hearing loss is related to various types of cognitive decline in both lab animals and humans [64]. However, underlying neuronal mechanisms causing perceptual and cognitive deficiencies still remain to be uncovered. Whereas the ability of sound localization in elderly subjects is generally degraded compared to young subjects [14,17,18,19], the degree of hearing loss itself is not a good predictor of localization performance [16], suggesting complex compensatory processes existing in the central auditory system. Along the auditory pathways of aged animals, loss of inhibitory transmissions is commonly observed [20,21]. In the present study, we used a binaural brainstem neuron model and investigated how its function is affected by a loss of inhibition.

Our simulations showed that the binaural tuning performances of the model neuron, which were measured by the modulation depth or the neuronal discriminability function, were reduced by up to 15% when a quarter of inhibitory inputs were eliminated (Fig 2). We then increased the amplitude of remaining inhibitory synaptic inputs to simulate compensatory effects against the loss of input fibers. With this input compensation, percentage of reduction in binaural tuning performances was suppressed to 10–25%, even when the number of inhibitory inputs was halved (Fig 3). These results indicate that binaural tuning of the LSO neuron is generally robust to moderate amount of inhibition loss, and that this robustness can further be enhanced by a recovery mechanism to counteract a more severe inhibition loss, possibly depending on a frequency-dependent manner (Fig 5). The relative paucity of physiological changes observed in aged LSO [24,26] might be explained by this robustness of binaural tuning. In order to test this hypothesis, future single-cell recordings in combination with histological counting of the numbers of synapses on the same cells will be required.

Existence of activity-dependent plasticity in the auditory brainstem was reported in the last decade [38,39]. And such plasticity may occur also in the population level to adjust the neuronal outputs of LSO neurons according to the input stimulus statistics [65]. Adaptive changes to stimulus statistics were also found in the midbrain inferior colliculus and are suggested to be a mechanism of robust binaural perception [66]. Whereas various types of homeostatic plasticity may exist in the real system on both cellular and network levels [33,34,35], we adopted a very simplistic form of compensation, in which all remaining synaptic inputs were uniformly amplified to keep the total synaptic conductance unchanged from the default (healthy) state. Furthermore, our simulation results in the overcompensated case (Fig 4) differed only slightly from the compensated case (Fig 3), suggesting that the exact amount of amplitude compensation may not have to be precisely controlled. Since these simple compensatory mechanisms were found effective in counteracting the loss of inhibitory inputs, we expect that more sophisticated forms of activity-dependent plasticity would render even further robustness to binaural tuning in the aging LSO. In young animals, the cell body and proximal dendrite of an LSO neuron are covered primarily by inhibitory terminals, while excitatory synapses are located more distally [1,67]. It would be a topic of future experimental and modeling studies how the distributions of synaptic location may or may not change with aging and how such morphological alterations may affect binaural coding.

### Degradation of temporal properties

In this study, we focused solely on the reduction of inhibitory synaptic inputs, assuming that the excitatory inputs were not significantly altered in the old LSO. This assumption needs to be tested in future anatomical and physiological studies. Experiments in human subjects using earplugs suggested the existence of subcortical auditory gain control mechanisms [68]. Animal studies also found an increased neuronal activity after noise damage in the cochlear nucleus (reviewed in [60]). These results imply that not only inhibitory inputs but also excitatory inputs might be homeostatically adjusted during aging. In aged animals including humans, the number of auditory nerve fibers is generally reduced [69,70]. The temporal precision of remaining fibers, however, does not differ from young animals [71]. Endbulb synapses between auditory nerve fibers and bushy cells in mice show compensatory adjustment of transmitter release (reviewed by [40]), which may contribute to the preservation of excitatory input to LSO. In the present study, we did not examine the effects of increasing temporal jitter in aged animals, since there are no physiological data currently available that can be used for calibrating or testing the modeled outcome. Reliability of spike timing is more likely to affect phase coding than level coding, as binaural phase-tuning in LSO requires temporally precise interaction of excitatory and inhibitory inputs [11]. Consistent with this hypothesis, ILD sensitivity both in behavior and in auditory midbrain neurons is robust to complete decorrelation of sound stimuli at the two ears [57]. Increase in temporal jitter degrades the synchrony of synaptic inputs, which would lead to a reduction of the modulation depth of phase-tuning curves (as demonstrated in [72], albeit in a different system). Influences of temporally imprecise inputs should be examined physiologically in the future, as there were no published recording results from the aged LSO that used dichotic AM sounds.

As reviewed in the Introduction, aging often leads to a reduction of the binaural interaction component (BIC) of the ABR, which is presumed to reflect the reduced efficacy of inhibition in LSO [30,32]. Our population modeling results, however, showed only minor changes in binaural ILD-tuning with reduced inhibition (Fig 7). This discrepancy might be explained by the required temporal precision of spiking responses. ILD-tuning depends predominantly on the spike rate differences of bilateral excitatory and inhibitory inputs, but not on the precise timings of these inputs. In contrast, prominent peaks in the ABR are likely to originate from the time-locked activity of many neurons, possibly including those responding only at the onset of sustained tonal stimulation [46]. We did not include such onset neurons in the present study due to the paucity of relevant physiological data for modeling. Nevertheless, we expect that BICs (as well as binaural phase coding) are probably more vulnerable to a reduced temporal fidelity of input neurons than ILD coding. This hypothesis is a subject of future physiological studies in both onset (principal) and sustained (non-principal) neurons of young and aged animals examined with transient stimuli, such as AM sounds and clicks (for a recent review, see [73]).

Previous behavioral studies demonstrated that aged human subjects show more difficulties in sound lateralization tasks relying on interaural time disparities than in those using level differences [74,75]. Our results suggested, however, that simulated tuning of phase difference is slightly more robust against the loss of inhibition than level difference tuning (e.g., Fig 2D and 2G). This discrepancy may be due to the difference in sound stimuli. We focused on the sustained responses of LSO neurons, while the above-mentioned behavioral studies used click trains that are highly transient in time. Furthermore, age-related functional changes in the medial superior olive, which is responsible for the detection of interaural time difference in the temporal fine structure of low frequency sounds [1,2], may also contribute to the altered binaural perception in elderly subjects (see [16] for related discussions).

### Sources of age-related degradation in binaural perception

Measurements of monaural and binaural ABRs suggest a general reduction of synchronized neuronal activity along the auditory pathways in aged animals [32]. Comparative studies of rodents, however, revealed that types and degrees of age-related changes in the auditory system may largely depend on animal species [76]. Even within the same species, the degree of age-related neuronal loss in MNTB can be different between genetic strains (e.g., more than 30% in Sprague-Dawley rats vs. less than 10% in Fisher-344 rats) [22,25]. Furthermore, only relatively recently was the existence of MNTB in humans conclusively confirmed [77]. It is unknown how much MNTB neurons are lost in elderly people and what kind of functional changes may occur in their binaural brainstem circuits.

Based on anatomical measurements in cats [78], we assumed that inputs to LSO (and MSO) from spherical and globular bushy cells in the anteroventral cochlear nucleus are driven primarily by high spontaneous rate auditory nerve fibers. In gerbils, age-related loss of high-threshold, low-spontaneous rate auditory nerve fibers was also observed [79]. Recent evidence in aged mice suggests that changes of low- and medium-spontaneous rate fibers may impact bushy cells’ function [80]. Furthermore, spiking activity of a bushy cell is modulated by inhibitory inputs [81,82] from several sources including D-stellate cells in the anteroventral cochlear nucleus [83], whose synaptic inputs were found to degrade with aging [84]. Activity-dependent adjustment of synaptic inputs may also occur at the level of bushy cells.

We used modulation depths and neuronal discriminability functions to estimate the performance of binaural processing in the model LSO. While these measures are supposed to reflect the encoding properties of LSO neurons, it is still unknown how the outputs of LSO neurons and other brainstem nuclei are combined in higher auditory areas that "decode" the binaural information to form the perception of sounds (see discussions in [7,85,86]). In our population modeling approach, we simply used the rate differences of mirrored LSO neuron pairs and averaged the corresponding discriminability functions within the simulated population (Fig 7). While the detailed decoding mechanisms in the downstream stages beyond LSO remain largely unknown, the use of discriminability allows us to compare theoretical bounds of precision for estimating the binaural cues [51]. This population averaging could be related to the integration of ILD cues in the midbrain [49,57]. Metrics such as marginal Fisher Information (e.g., [87]) might be used to examine which stimulus dimensions can no longer be encoded by single or populations of neurons in aged animals.

In our comparisons across different number of inhibitory inputs, we took a population average of the discriminability and normalized it to the default condition (Fig 7). This average/normalization procedure was used to eliminate the effects of the population size and other factors, such as the recording time length, that affect the trial-to-trial variability in spike rates. Currently there are no behavioral or neurophysiological data available that can be compared directly with our simulated population discriminability. In a future comparison between theoretical and experimental data, unnormalized discriminability could also be used for examining the actual amount of trial-to-trial variability and the robustness of binaural coding in the real system. Our population modeling assumed symmetric changes in the LSO of both sides, whereas hearing loss of a various extent can often happen asymmetrically between the two ears [88]. To more rigorously characterize the effects of age-related loss of synaptic inputs at the brainstem level, further experimental knowledge on physiological functions of the decoding stages will be important.

In combination with prior physiological observations [24,26], our computational results suggest that the main source of age-related decline in binaural perception may originate from higher auditory stages than LSO. In the midbrain inferior colliculus, which are projected directly and indirectly from the binaural nuclei in the brainstem [2], many fundamental response properties remain relatively unaffected during aging, while the representation of external acoustic environment is deteriorated possibly due to the decrease of temporal processing fidelity (reviewed in [89]). Neurons in the midbrain superior colliculus, which receives inputs both from the inferior colliculus and from the visual system, present degraded auditory spatial selectivity in aged guinea pigs [90] and aged rats [91]. Auditory cortical neurons in aged monkeys also present broader spatial tuning than in young monkeys, which is probably associated to age-related reduction of inhibitory inputs [92]. Contribution of higher auditory stages is supported also by the observation that binaural discrimination performance in aged subjects is associated with cognitive performance [93]. Future systematic comparisons across auditory areas will thus be necessary for identifying the relative contribution of each stage along the entire central auditory pathway to the age-related decline of binaural perception.
